# Mothers’ and fathers’ sense of security in the context of pregnancy, childbirth and the postnatal period: an integrative literature review

**DOI:** 10.1186/s12884-018-2096-3

**Published:** 2018-12-04

**Authors:** Therese Werner-Bierwisch, Christiane Pinkert, Karin Niessen, Sabine Metzing, Claudia Hellmers

**Affiliations:** 1 Faculty of Health, Department of Nursing Science, Witten/Herdecke University, Stockumer Strasse 12, 58453 Witten, Germany; 2 Research Group `FamiLe – Family Health in Life Course`, Witten and Osnabrück, Germany; 3 Faculty of Business Management and Social Sciences, Osnabrück, Osnabrück University of Applied Sciences, Germany, P.O. Box: 1940, 49009 Osnabrück, Germany

**Keywords:** Sense of security, Mothers, Fathers, Pregnancy, Childbirth, Postnatal period, Literature review

## Abstract

**Background:**

From the individual perspective, security, which is essential to life quality, is characterised as an elementary human need that requires fulfilment. During the transition to parenthood, mothers and fathers are confronted with changes in physical and psychosocial processes that are accompanied by uncertainty and insecurity. Feelings of insecurity may have consequences affecting their pregnancy and childbirth experiences as well as their adaption to the parental role in the first weeks following childbirth. In this context, it is important to understand how parents express and interpret their sense of security to effectively support their security needs. This integrative review aimed to provide a critical synthesis of existing research on parents’ experiences of their sense of security associated with pregnancy, childbirth and the postnatal period.

**Methods:**

A literature search of the PubMed, CINAHL, PsycINFO and GESIS Sowiport databases was performed. Peer-reviewed papers that were published in English or German between 1990 and 2017 focusing on mothers’ and fathers’ experiences of sense of security in the context of maternity care were included. A thematic analysis was performed to organise and describe the findings.

**Results:**

Eleven research-based papers met the inclusion criteria. Four key themes among the data were analysed: the meaning and manifestation of sense of security, sense of security in relation to confidence and control, lack of feeling secure and coping strategies, and factors influencing sense of security.

**Conclusions:**

The findings revealed a complex profile of the perception of security associated with pregnancy, childbirth and the postnatal period. Sense of security can depend on multiple internal and external factors, which can differ between mothers and fathers. Research on the experiences and perceptions associated with fathers’ sense of security is lacking. Further research focused on the experiences of security from the parents’ perspective is necessary. Midwives and other involved health professionals should be aware of their role in creating a sense of security among parents. Based on a local specific understanding of security experiences, professional caregivers have the opportunity to support parents more effectively with regard to their specific security needs.

## Background

Security is an essential part of the quality of life [1]. As a need required by all humans since the beginning of mankind, security affects nearly all aspects of human life and is therefore considered an “anthropological constant” [2]. In the transition to parenthood, expectant parents are exposed to personal, familial, and social changes [3, 4], which have important implications for the couple’s relationship, the infant-parent relationship and the infant’s development [4, 5]. The reorganisation and preparation of new living conditions and new social roles may be accompanied by subjective uncertainties, insecurities and anxieties [4, 5]. During pregnancy, a woman’s physical changes can be perceived as very positive and contribute to great pride in the abilities of one’s own body. On the other hand, these changes can be perceived as threatening because of the uncontrollable process, which can lead to a sense of insecurity and increase childbirth-related fear [6]. For expectant fathers, pregnancy can be accompanied by great joy, and thus a high degree of paternal involvement in pregnancy processes [7, 8], or by uncertainties and anxieties regarding the paternal role during pregnancy and childbirth [9–12]. Perceived insecurities associated with pregnancy and childbirth may have serious consequences regarding the childbirth experiences of mothers and fathers, their adaption to the parental role, and their attachment to the child during the postnatal period.

Security is a familiar term to most people; however, the term security has multiple meanings, and the intended meaning is not always clear [2, 13–15]. The definition of security is imprecisely differentiated from similar terms, such as safety [16, 17], certainty and dependability [2, 18–20], making finding a common basis for discussion difficult. A feature shared by most definitions is the distinction between objective and subjective dimensions of security. Objective security is described as an absence of threats [15, 17, 19, 21] and is accompanied by risk assessments, as lower risks are associated with a higher objective security [22, 23]. However, objective security does not exist separate from the individual’s perception [24], and the subjective perception of security can thus deviate from the objective state of security [15, 19, 21, 24]. Therefore, this review begins with a theoretical discussion on the sense of security concept.

### The sense of security concept

From the perspective of the individual, security is characterised as an elementary human need that requires fulfilment [19, 25–27]. Individuals strive for security to maintain the ability to act and to minimise the risk of being paralysed and unable to act [19]. In this process, decisions are made with the aim of producing the desired future results and thus reducing future insecurities [2, 27, 28].

Sense of security is characterised by cognitive and affective components [24, 29]. On the one hand, sense of security is understood as a perception and thus represents a cognitive construction of the individual [10, 14, 17], which includes the perception of a physical state with the sense of being protected against threats [2, 19, 20, 24, 30]. On the other hand, sense of security is commonly expressed as an individual emotion in which a person feels safe [16, 17], free from anxiety [15, 19, 21], carefree [2, 19, 29], confident [19, 20, 30] and without doubt [2, 19].

Feeling secure or insecure depends on diverse internal and external factors. The factors defining sense of security are an individual’s perception of being vulnerable and at risk in addition to an assessment of one’s own coping ability [2, 28, 30]. Gender, age, past experiences, personality traits, and information may also play roles in the creation of security [28, 31, 32]. Social science studies demonstrate a partial mismatch between the objective security of a given situation and the sense of security [20, 28, 30, 31, 33]. An individual can feel secure despite the presence of objective danger because the danger is not recognised or perceived as such [19]. On the other hand, a person may feel secure despite an objective danger due to perceived coping abilities [19, 28, 30] or self-confidence [19, 20]. However, the subjective perception of security is strongly dependent on the context; therefore, the specific factors are relative and not equally relevant in all spheres of life [32].

Analysis of the social science literature shows that no clear dividing line exists between feeling safe and feeling secure; both terms are often used synonymously, indicating that the concepts of security and safety are interwoven [16]. Bar-Tal and Jacobson [24] argue that maintaining physical safety is a prerequisite for a subjective perception of security, which they consider to be a psychological need.

In the context of maternity care, the feeling of security is described as one of the elementary aspects of a mother’s overall birth experience [34–38] and, for example, plays an important role in the maintenance of breastfeeding during the postnatal period [39]. According to Mozygemba [6], women strive for security during pregnancy and birth to orientate themselves during the processes of change. Further studies focusing on childbirth experiences indicate that the presence and professional support offered by midwives and hospital staff contribute to creating a sense of security for mothers [40–44] and fathers [43, 45–48]. Additionally, the partner’s presence at the delivery is considered beneficial for the women’s sense of security [49–51]. Moreover, absence of the midwife from the delivery room or not knowing how to support the woman can create a sense of insecurity for the father [43, 52].

Ideas regarding what contributes to the feeling of security can differ between the supporting midwives and the parents; while women and their partners consider the midwife’s presence important to their sense of security, the midwives do not explicitly describe their own presence as supporting the parents’ sense of security during labour [43].

### Safety and risk concepts in the obstetric context

In most Western societies, the contemporary childbirth culture is embedded in concepts of safety and risk. In this context, the concept of safety represents a quality feature of maternity care, as it aims to reduce the risk of unnecessary harm for mothers and their babies to an acceptable minimum [53]. The declaration of the concept of risk as “the key to safety” [54] results in understanding pregnancy, childbirth and the postnatal period as potential risks [55, 56], which fosters the medicalisation of pregnancy and childbirth [57–59]. It appears that the concepts of safety and risk complement one another and have a restrictive effect on giving birth. In this context, parents are confronted with rules and normative views of a maternity healthcare system that influence their attitudes, decisions and actions.

Studies regarding the perception of safety in the context of maternity care indicate that the safety concept is differentially perceived and interpreted. For some women, safety is associated with medical-technical care and thus also with risk minimisation [60–63]. For other women, being safe means avoiding routine interventions, giving birth in a calm and private atmosphere, having confidence in the ability to give birth or having trust in God [61, 62]. The various interpretations of safety are associated with different attitudes towards childbirth and the competence of the female body to perform birth. On the one hand, pregnancy and childbirth are understood as fundamental medical processes that require medical skills and technology [60, 61, 63, 64]; on the other hand, they are perceived as natural processes that do not require medical interventions or professional assistance [61–63]. In addition, the perception of safety can be influenced both negatively and positively by the care offered by midwives and other professionals [65–67]. The birth experience itself, including the experience of pain and the birth of a healthy child, can also retrospectively influence a woman’s view of safety [68]. Further studies demonstrate that the various perceptions of safety have a decisive impact on women’s choice of where to give birth, such that both the hospital and the home are considered safe birthplaces for women [60–63, 66, 69, 70].

Research has revealed different meanings of safety and different needs of women. The results underline that women’s perception of safety goes beyond the medical understanding and is influenced by other cultural, emotional and psychosocial aspects.

We acknowledge the existence of a possible overlap between the concepts of safety and security. Furthermore, the concepts of safety and risk in maternity care presumably influence the sense of security.

### Formulation of the problem

Theoretical and empirical literature characterise subjective perceived security as an elementary human need that requires fulfilment. During the transition to parenthood, mothers and fathers are confronted with physical and psychosocial changes that are accompanied by uncertainty and insecurity. Feelings of insecurity may have consequences for parents’ pregnancy and childbirth experiences, adaption to the parental role, and attachment to the child.

However, no study to date has critically synthesised the research literature regarding sense of security from the perspective of parents in the context of maternity care. This paper presents an integrative review of the literature addressing parents’ sense of security associated with pregnancy, childbirth and the postnatal period. For the purposes of this paper, sense of security has been defined as an emotional state in which an individual feels safe, confident and free from doubt and anxiety. A critical synthesis of current knowledge regarding parents’ security experiences in the context of maternity care is important for understanding and effectively supporting mothers and fathers with regard to their specific security needs.

## Methods

### Aim

This review aimed to provide a critical synthesis of research concerning parents’ experiences of sense of security in association with pregnancy, childbirth and the postnatal period. The following research questions guided this review:

1. How do parents experience a sense of security in the context of pregnancy, childbirth and the postnatal period, and how does it manifest?

2. Which factors influence parents’ sense of security?

### Methodology

The integrative literature review method is considered the most appropriate method for exploring the status of available literature with diverse methodologies [71]. This literature review is based on Whittemore and Knafl’s [71] five-step recommendation: formulation of the problem, literature search, evaluation of data, data analysis, and presentation of the results.

### Literature search

A three-step screening process was used to identify relevant publications. Step one involved a broad search of the electronic databases PubMed, CINAHL, PsycINFO and GESIS Sowiport. Studies published in English or German between January 1990 and April 2017 were explored. The decision to start the literature search in 1990 was based on the assumption that medical-technical care and increasing intervention rates beginning in the 1990s may have impacted the sense of security concept. In addition to the database search, a manual search of the reference lists of relevant publications was performed. Both German and English search terms were used, and the keywords security, sense of security, feeling safe‚ experience, perception, pregnancy, labour and delivery, childbirth, postnatal period, postpartum period and postpartum care, were used in various combinations with medical subject headings (MeSH) and free text words.

**Fig. 1:**
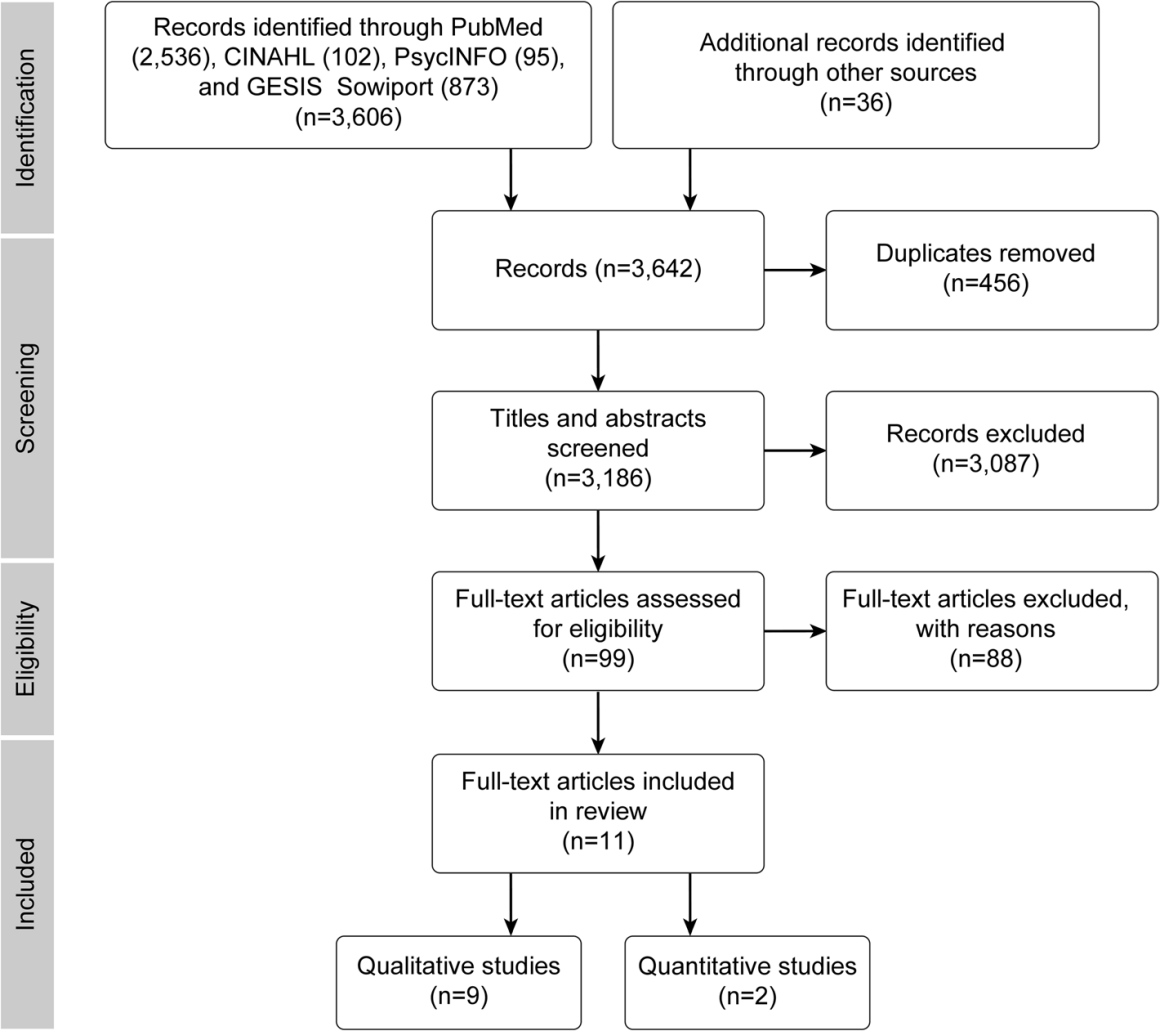
PRISMA flow diagram of the search strategy outcomes

The literature search (Fig. 1) identified 3606 potential articles from the databases and an additional 36 studies from the reference lists. In the second step, after the exclusion of duplicates, 3186 titles and abstracts were screened for relevance according to the research questions. Third, of the remaining 99 full-text articles, manuscripts were included only if they focused on mothers’, fathers’ or parents’ experiences of sense of security during pregnancy, childbirth and the postnatal period.

To obtain a comprehensive profile of sense of security, studies were also included if the phenomenon was described as one of several results in the context of pregnancy and childbirth experiences. Studies in which the views of mothers/fathers were combined with those of professionals in the results analysis as well as studies focusing on views from the perspectives of nurses, midwives and obstetricians were excluded. A total of 11 research reports met the inclusion criteria, including nine qualitative studies and two studies that utilised a quantitative approach. The first author searched databases and manually searched reference lists, screened relevant titles and abstracts, and identified potential papers for inclusion. The inclusion of potential papers was discussed with two other authors.

### Evaluation of data

The scientific rigour of the included studies was appraised using checklists provided by Critical Appraisal Skills Programme (CASP) tools. The validity and quality of the qualitative studies were assessed using the CASP Qualitative Checklist [72]. Analogous to the CASP Qualitative Checklist, the included quantitative studies with a cross-sectional design were assessed with the same critical appraisal method using the CASP Cohort Study Checklist [73]. While the evaluation criteria of the CASP Cohort Study Checklist are highly similar to those of cross-sectional checklists [74], the scope is smaller and more manageable. Critical appraisal of all included studies was performed by the first author. Of these studies, five were appraised independently by a second researcher to ensure consistency in the appraisals between researchers. Some of the included studies did not meet all the qualitative criteria but provided important insights into the sense of security phenomenon. Therefore, no studies were excluded after the appraisal process. The appraisal details of the critical evaluation are presented in Table 1.

**Table 1:** Appraisal of studies by study design using CASP tools

Qualitative studies (Qualitative Checklist)											
Author(s)	Was there a clear statement of the aims of the research?	Was a qualitative methodology appropriate?	Was the research design appropriate to address the aims of the research?	Was the recruitment strategy appropriate to address the aims of the research?	Was the data collected in a way that addressed the research issue?	Was the relationship between the researcher and participants adequately considered?	Were ethical issues taken into consideration?	Was the data analysis sufficiently rigorous?	Was there a clear statement of findings?	Is the research valuable?	
Côté-Arsenault and Donato [76]	Y	Y	Y	Y	N	N	N	Y	Y	Y	
Ekström et al. [85]	Y	Y	Y	N	Y	N	Y	Y	Y	Y	
Halldorsdottir and Karlsdottir [77]	Y	Y	Y	N	Y	N	Y	Y	Y	Y	
Karlström et al. [78]	Y	Y	Y	N	Y	Y	Y	Y	Y	Y	
Melender and Lauri [79]	Y	Y	Y	N	Y	N	Y	Y	Y	Y	
Namey and Lyerly [81]	Y	Y	Y	N	Y	N	Y	Y	Y	Y	
Persson and Dykes [83]	Y	Y	Y	N	Y	Y	Y	Y	Y	Y	
Persson et al. [82]	Y	Y	Y	Y	Y	N	Y	Y	Y	Y	
Persson et al. [87]	Y	Y	Y	Y	Y	Y	Y	Y	Y	Y	
Quantitative studies (Cohort Study Checklist)											
	Did the study address a clearly focused issue?	Was the cohort recruited in an acceptable way?	Was the exposure accurately measured to minimise bias?	Was the outcome accurately measured to minimise bias?	Did the authors identify all important confounding factors? Did they consider the factors in the design and/or analysis?	Was the follow-up of subjects sufficiently complete/long enough?	Were the results presented transparently and precisely?	Are the results plausible?	Can the results be applied to the local population?	Do the results of this study fit with other available evidence?	Does the study have implications for practice?
Melender and Lauri [80]	Y	Y	Y	Y	NA	Y	Y	Y	Y	Y	Y
Persson and Dykes [84]	Y	Y	Y	Y	NA	Y	Y	Y	Y	Y	Y

*Y* yes, *N* no, *NA* not answered

### Data analysis

The 11 remaining studies were synthesised under the following subheadings and recorded on a data-coding sheet: author and country, objective, study sample and inclusion criteria, research design, data collection method(s), instrument(s), method of analysis, key findings regarding experiences of sense of security and limitations (Table 2).

**Table 2:** Synoptic view of papers included in the review

Qualitative studies						
Authors and country	Objective	Study sample and inclusion criteria	Research design and data collection method(s), instrument(s)	Method(s) of analysis	Key findings	Limitations
Côté-Arsenault and Donato [76], USA	To describe women’s late pregnancy after loss experiences	Purposive sampling; *n* = 69 pregnant women	Qualitative, longitudinal study Pregnancy calendar entries and field notes provided from 10 to 17 weeks gestation to birth	Thematic analysis	Previous experiences influenced the sense of security in the subsequent pregnancy. Assessing foetal movements and regular feedback from a care provider contributed to a sense of security.	Relatively unstructured and extensive data collection
Ekström et al. [85], Sweden	To explore fathers’ feelings and experiences during pregnancy and childbirth	Purposive sampling; *n* = 8 first-time and experienced fathers	Qualitative, inductive approach Written interviews (data collection after birth)	Content analysis	Being prepared to welcome the baby gave first-time fathers a sense of security. The confidence in healthcare professionals contributed to paternal sense of security. The lack of control over the beginning of the childbirth created insecurity.	Recruitment by midwives at two maternity wards Small sample size Only five of twenty fathers returned their written interviews Data from the pilot study was included in the analysis
Halldorsdottir and Karlsdottir [77], Iceland	To explore the essential structure of the experience of childbearing from the perspective of women	Purposive sampling; *n* = 14 mothers	Qualitative, phenomenological approach Interactive interviews	Thematic analysis	Feeling safe was perceived as a need while in labour and delivery. Fulfilment through the support and presence of a midwife and the partner reduced fear and encouraged admittance to the birth.	Small sample No statement about the period of data collection The results refer to a limited geographic area, and thus, a cultural and social group of participants was represented in this study
Karlström et al. [78], Sweden	To describe women’s experiences of a very positive birth experience	Purposive sampling; *n* = 26 first-time and experienced mothers	Qualitative (part of a prospective longitudinal cohort study) Focus group discussions (6–7 years after giving birth)	Thematic analysis	Feeling safe was an essential part of positive birth experiences and was confirmed by the presence of the child’s father, a trustful relationship with the midwife, and a positive atmosphere in the birthing suite. Feeling safe was essential to gaining control during birth.	The timing of recruitment: 6–7 years after the index birth Relatively homogenous sample group
Melender and Lauri [79], Finland	To describe security associated with pregnancy and childbirth	Convenience sampling; *n* = 20 Finnish women (primiparous and multiparous) during pregnancy	Qualitative Semi-structured interviews (23–36 weeks of gestation)	Content analysis	The elements creating security associated with pregnancy and childbirth were maternity healthcare, social support, a sense of control and one’s own attitudes. If the feeling of security was lessened during pregnancy, a search for support was performed through the women’s social network and contact with the midwife or obstetrician.	The sampling strategy is insufficiently described
Namey and Lyerly [81], USA	To deconstruct the term “control” as used by childbearing women	Purposive sampling; 101 mothers *n* = 39 (primiparous women) and *n* = 62 (multiparous women) who had given birth in various birthplaces and experienced different modes of delivery	Qualitative Semi-structured interviews (between April 2006 and July 2009)	Data analysed using NVivo software. Method of key word concept analysis by Quinn	Part of the definition of control relates to personal security, which encompasses feelings of physical safety and emotional and psychological attributes of security, such as comfort and confidence in one’s surroundings. Management of the birth experience and minimisation of anxiety or fear are associated with feelings of personal security.	Sample: Only parous women Recruitment primarily from a defined geographical area The recruitment method was held constant across the predefined sampling characteristics
Persson and Dykes [83], Sweden	To reveal factors that influence the experiences of mothers and fathers when they choose to return home	Purposive sampling; *n* = 12 (first-time and experienced) parents	Qualitative. Grounded theory Open interviews (2–3 weeks after giving birth)	Coding by Strauss and Corbin	The most important factors for creating a sense of security for the parents were the midwife’s empowering behaviour, affinity within the family, autonomy and sense of control and physical well-being.	Purposive recruitment from one hospital by using theoretical sampling strategy Restricted variety of experiences by only healthy participants
Persson et al. [82], Sweden	To describe factors influencing mothers’ sense of security during the first postnatal week	Purposive sampling; n = 14 mothers	Qualitative descriptive design Open interviews and focus group discussions (2–11 weeks after giving birth)	Thematic content analysis by Burnard	Mother’s postnatal sense of security was dependent on support from staff, support from the partner, and the mother’s and baby’s physical health and well-being.	Variation in time span for conducting the interviews Participants with lower educational levels were under-represented
Persson et al. [86], Sweden	To explore and describe factors that influence fathers’ sense of security during the first postnatal week	Purposive sampling; *n* = 13 fathers	Qualitative Open interviews and focus group discussions (3–9 weeks after childbirth)	Thematic content analysis by Burnard	Participation in the processes of pregnancy, birth and early parenthood fostered fathers’ postnatal sense of security. Fathers felt secure when they had someone knowledgeable to ask and when they were heard and taken seriously.	Variation in time span for conducting the interviews
Quantitative studies						
Authors and country	Objective	Study sample and inclusion criteria	Research design and data collection method(s), instrument(s)	Method(s) of analysis	Key findings	Limitations
Melender and Lauri [80], Finland	To describe elements creating a sense of security associated with pregnancy and childbirth, the manifestation and the influence of background factors	*n* = 329 (primiparous and multiparous) women who were 16–40 weeks pregnant; 69% response rate	Quantitative, descriptive design Structured questionnaire Development of the instrument was based on a pilot study and review of the literature	Factor analysis, Kruskal-Wallis test, and Mann-Whitney U-test	*Elements that created a sense of security* 1. Support from relatives (REP 12.4%) 2. Knowledge about pregnancy, childbirth and childcare (REP 10.5%) 3. Prenatal healthcare experiences and support from healthcare professionals (REP 8.8%) 4. Support from the partner (REP 8.5%) 5. Livelihood (REP 7.6%) 6. Positive stories heard about pregnancy, childbirth and baby care (REP 7.5%) *Elements creating security in relation to background factors* Knowledge about pregnancy, childbirth and childcare was reported significantly more often by experienced mothers. Prenatal healthcare experiences create security significantly more often in women without pregnancy-related problems compared to those with such problems. Support from the partner was reported significantly more often by women without pregnancy-related problems than those with such problems.	Limitations were not stated
Persson and Dykes [84], Sweden	To evaluate dimensions of both parents’ postnatal sense of security during the first week after childbirth	*n* = 113 mothers and *n* = 99 fathers of every fifth baby born live at term at five hospitals in southern Sweden; 71% response rate (mothers) and 63% response rate (fathers)	Evaluative, cross-sectional design Postal questionnaire consisted of background questions, parents’ postnatal sense of security (PPSS) instrument and State Trait Anxiety Inventory (STAI) trait instrument (8 weeks postpartum)	Mann-Whitney U-test and multiple regression analysis	First-time mothers felt significantly less postnatal security than experienced mothers. A sense of midwives’ empowering behaviour and a sense of personal well-being (for mothers) were significantly associated with security. For mothers, parity was significantly associated with security together with a sense of participation in care, a sense of the partner’s participation during pregnancy, expected positive childbirth and security experienced during birth. For fathers, a sense of participation during pregnancy was significantly associated with security.	There was no analysis of the dropouts due to the study design

*REP* relative explanatory power

There is no gold standard for the analysis of data in an integrative review [71]. The method used for the interpretation of data in this review was a thematic analysis, which is a flexible method for identifying, analysing and reporting prominent themes within data [75] and a means of integrating qualitative and quantitative evidence. To accomplish the aim of this review, themes were initially identified from the results of each study, and patterns, similarities and differences among the included articles were then defined. In the second step, factors that influence sense of security were coded in a deductive manner to answer the research question. The identified themes were then discussed among authors.

## Results

The studies selected for this review used qualitative (*n* = 9) and quantitative (*n* = 2) methodologies. The majority of the included studies were based on interviews with pregnant women or mothers [76–82]. In two studies, the mothers were interviewed together with their partners [83, 84]. Only two relevant studies focusing on the perspective of the fathers were identified [85, 86].

Sense of security received different degrees of attention in the included studies and was expressed in both similar and different terms. In some studies, security and safety were used synonymously without an exact description [76]. Further studies used the terms *feeling safe*, *feeling secure*, *feeling safe* and *secure* [77, 78, 85], *sense of safety* [83], or *personal security* [81]. Most studies used the term *sense of security* [79, 80, 82–84, 86], where this concept was described by the authors and coincided with the essential points of the definition used in this review [79–82, 86].

Four key themes identified from the data were analysed: the meaning and manifestation of sense of security, sense of security in relation to confidence and control, lack of feeling secure and coping strategies and factors influencing sense of security.

### Theme 1. The meaning and manifestation of sense of security

The feeling of security was characterised as a mother’s essential need while giving birth [77] and as a central issue for women during pregnancy and childbirth or for parents in the first week after birth [76, 83]. Karlström et al. [78] reported that feeling safe and secure was an essential part of women’s positive birth experience. Perceptions of what feels safe influenced the mothers’ behaviours and decisions at any time during pregnancy, birth and the first postnatal weeks [76, 79, 83, 84].

Melender and Lauri [79, 80] investigated how the feeling of security manifests in women. Security is revealed in one’s own resources and in feelings of comfort, calmness, joy, positive self-awareness, satisfaction and confidence that everything will go well. Everyday life (without focusing exclusively on pregnancy) has been described as a manifestation of security. With regard to pregnancy, an orientation towards the child and home preparation for birth are perceived as expressions of security by women [79]. Halldorsdottir and Karlsdottir [77] reported that during the childbirth journey, security manifested in a ‘go with the flow’ attitude towards the body and as a companion to the birth pains upon fulfilment of the need for security by a caring midwife and a present partner.

Regarding the experience of fathers, sense of security is described as an important factor influencing their decisions and behaviour during the postnatal period [83]. The importance of feeling secure is expressed in the desire of fathers’ participation during pregnancy, childbirth and the early postnatal period and in the need to be prepared for the baby, birth and fatherhood [83–86]. Fathers’ participation in care has a positive effect on mothers’ postnatal sense of security [84].

### Theme 2. Sense of security in relation to confidence and control

Relevant studies have indicated an association between sense of security and feelings of control and confidence [77–79, 81, 83]. Women who have confidence in themselves, their body and their ability to give birth feel secure during pregnancy or in the childbearing process and describe their birth experiences very positively [78]. The promotion of security during care leads to a reduction in anxiety and is conducive to a sense of control during the birth experience [78, 79]. Namey and Lyerly [81] describe personal security as part of the control concept, as it allows women to cope with the birth situation while minimising anxiety. Persson et al. [82] draw the same conclusion, revealing that reduced anxiety, positive feedback and confirmation of normal development during pregnancy can strengthen body confidence and increase mothers’ sense of security.

A subtle reference to a relationship between fathers’ feelings of security and confidence is reported in only the manuscript published by Ekström et al. [85]. The perceived competence of healthcare professionals contributes to the confidence that fathers have in caregivers and creates a sense of security.

### Theme 3. The lack of feeling secure and coping strategies

The implied inverse connection of sense of security, confidence, feeling of control and lack of anxiety with sense of insecurity, lack of trust, lack of control and feelings of anxiety has been demonstrated. In their qualitative study, Halldorsdottir and Karlsdottir [77] concluded that when women lose their sense of control due to a perceived lack of caring or lack of security, they can develop feelings of helplessness and perceive their birth as an unbearable experience. Côté-Arsenault and Donato [76] concluded that previous loss experiences have negative impacts on the sense of security in subsequent pregnancies; the sense of security in these situations is often described as fragile and can be rapidly undermined by problems emerging during pregnancy. Regular responses from the baby in the form of foetal movements, more frequent prenatal visits and ultrasounds, and positive feedback from the care provider contribute to a sense of security by providing as much data as possible [76]. Melender and Lauri [79] reported similar results regarding coping strategies. The authors concluded that if the sense of security decreases during pregnancy, women first attempt to cope by searching for social support and talking with their partner before seeking contact with healthcare providers. This coping strategy is based on the intensification of contact with the individual’s partner and medical care providers to maintain a confirmation of normality [76, 79].

For fathers, the lack of control over the beginning of the birth creates insecurity, and fathers may feel excluded and dependent on information from the mother [85]. In addition, anxiety over not arriving to the delivery ward in time or even having to deliver the baby themselves creates feelings of insecurity, which result in doubts about one’s own abilities [85]. The included studies did not investigate fathers’ strategies for coping with subjective perceived insecurity.

### Theme 4. Factors influencing sense of security

Melender and Lauri [79, 80] made major contributions to the description of sense of security associated with pregnancy and childbirth. In their qualitative study [79], women described the following elements associated with feelings of security: pregnancy care and related positive experiences, social support (particularly by the partner), and information on pregnancy, childbirth, child care and women’s lifestyle. In a subsequent quantitative study, Melender and Lauri [80] assessed the elements that create security and the influence of socio-biographical factors on the feeling of security. Security-promoting elements (social support from the family, knowledge about pregnancy, positive nursing experience, partner support, financial situation and positive experience with multiple children) are positively assessed by women as a whole. Sociobiographical factors also affect the security experience, as women with no pregnancy problems feel significantly more secure than women with pregnancy problems, and similar observations have been made between experienced mothers and first-time mothers [80]. Regarding parity, Persson and Dykes [84] reached a similar conclusion that first-time mothers feel significantly less secure than experienced mothers.

Persson and Dykes [83, 84] investigated a common perspective of parents related to the sense of security during the postnatal period. A qualitative study [83] demonstrated that positive, empowerment-motivating behaviours promoted by midwives, affinity towards family, physical well-being and autonomy are the most important factors for creating a sense of security during the first postnatal week.

For fathers, continuous participation during pregnancy is significantly associated with feelings of security [84]. The results of a further qualitative study performed by Persson et al. [86] regarding fathers’ security experiences support the previous findings and indicate that fathers’ postnatal sense of security emerges from participation in and experiences of the pregnancy, childbirth and early parenthood processes. The authors conclude that aspects of antenatal care are influencing factors that contribute to the feeling of security. Fathers’ participation in the pregnancy and childbearing processes is based on self-motivation and achieved via participation in parental education meetings or antenatal visits and dialogue with friends [83, 85, 86]. However, participation is essentially dependent on the support of the healthcare professionals and women themselves. Fathers feel secure if they have a sense of participation during pregnancy and decision making during birth [83, 84, 86], have a feeling of being taken care of during childbirth [85, 86] and are heard and taken seriously [86]. Persson et al. [82] concluded that fathers do not regard the postnatal weeks as an isolated time but rather as a phase in the parenthood process that indicates a dependency of the feeling of security on the time of pregnancy and birth and participation in the processes.

Most of the included studies contributed to answering the second research question and presented a broad spectrum of internal and external factors influencing parents’ sense of security. The internal factors of mothers (Table 3) can be summarised in the following groups: emotional states, knowledge/experiences, and health states of the mother and child. The external factors consist of attributes and acts of supporting persons, settings and options of maternity care and personal life situations. For fathers, the health states of the mother and child are considered external factors. Summaries of the factors influencing mothers’ and fathers’ sense of security are presented in Tables 3 and 4, respectively.

**Table 3:** Factors influencing mothers’ sense of security

Internal factors	Emotional state, knowledge and experiences
	─ Sense of control [78–81]
	─ Autonomy [81]
	─ Comfort [81]
	─ Feeling of not being alone [79]
	─ Confidence in ability to give birth [78, 81, 83]
	─ Confidence in one’s surroundings [81]
	─ Positive attitudes to pregnancy and childbirth [79, 80]
	─ Knowledge about pregnancy, childbirth and baby care [79, 80]
	─ Positive stories about pregnancy, childbirth and baby care [79, 80]
	─ Previous childbirth experiences [79, 80, 82, 84]
	Physical health of mother and child
	─ Foetal movements and growth [76, 79, 80]
	─ Physical well-being of mother and child [79–84]
	─ Pregnancy-related complications [79, 80]
	─ Active self-care and care of unborn child [76]
External factors	Attributes and acts of supporting persons
	─ Presence of a caring midwife [77–79]
	─ Support of a caring midwife when needed [77, 78]
	─ Trustful relationship with a caring midwife [77–79]
	─ Support/regular feedback from healthcare professionals [76, 79, 80]
	─ Being seen as an individual [82]
	─ Consistent advice/information [77, 78, 82]
	─ Partner’s and own participation in care [82, 84]
	─ Presence and support of the partner [77–80, 82, 83]
	─ Support from relatives [80]
	Settings and options of maternity care
	─ Prenatal visits and ultrasound [76]
	─ Medical expertise/maternity healthcare [76, 79]
	─ Positive atmosphere in the birthing suite [78]
	─ Avoiding interventions [81]
	─ Planned follow-up after birth [82]
	Personal life situation
	─ Livelihood [79, 80]

**Table 4:** Factors influencing fathers’ sense of security

Internal factors	Own emotional state, knowledge and experiences
	─ Being prepared to welcome the baby [83, 85]
	─ Lack of control over the beginning of birth [85]
	─ Concern about not arriving at the delivery ward in time and having to deliver the baby themselves [85]
	─ Confidence in healthcare professionals [85]
	─ Sense of participation during pregnancy [84, 86]
	─ Sense of participation in decision making during birth [83, 84, 86]
	─ Sense of being responsible for the care of the mother and the child at home [83, 86]
	─ Knowing who to ask [86]
	─ Previous experiences of childbirth [85, 86]
	─ Affinity to mother of the common child [81]
External factors	States of health of mother and child
	─ Emotional well-being of the mother [84]
	─ Physical well-being of the baby/ability to breast feed [83, 84, 86]
	─ Birth without complications [85, 86]
	─ Woman’s experiences and knowledge [85, 86]
	Attributes and acts of supporting persons
	─ Competence of healthcare professionals/midwives [85, 86]
	─ Midwives’/nurses’ empowering behaviour [83, 84]
	─ Cooperation with the midwife [85]
	─ Participation in the care of the baby [83, 86]
	─ Information from friends and midwives [85]
	─ Being taken care of and attention during childbirth [85, 86]
	─ Being heard and taken seriously [86]
	─ Being given confirmation about the normality of the situation [86]
	─ Being given relevant and consistent information and explanations [86]
	Settings and options of maternity care
	─ Participation in antenatal visits [83, 86]
	─ Parental education meetings [85]
	─ The opportunity to be together with the mother and child at hospital [86]
	─ Postnatal visits at home [83]
	─ Available medical resources after childbirth [83, 85]
	Personal live situation
	─ Returning home after childbirth [86]
	─ Possibility of being together with the mother and child at home [86]

## Discussion

The aim of this literature analysis was to examine the phenomenon of sense of security from mothers’ and fathers’ perspectives in the context of pregnancy, childbirth and the postnatal period. The results demonstrate a broad range of factors influencing the feeling of parental security. For women, the attribution of security as a basic need and central issue in pregnancy, childbirth and the postnatal period represents an important result of the literature analysis and corresponds to sociopsychological findings [19, 24–27]. The pursuit of security during pregnancy, childbirth and the postnatal period is an essential feature that can be regarded as a process characterised by continuous negotiations.

The findings of the analysis suggest a correlation between sense of security and feelings of control and confidence. Being in control during childbirth is an important factor for a woman’s birth experience and her well-being [87–90]. Because “control” has been differentially conceptualised in previous research [91], exactly how the control concept is connected with sense of security is not completely understood. Confidence in the ability to give birth is congruent with a woman’s self-efficacy for coping with the birth situation [92]. However, it remains unclear whether the sense of security is a cause or a consequence of the sense of control and confidence.

The association between feeling secure and being free from anxiety is theoretically reflected in various references [15, 19, 21]. Analogically, the results implied an association of sense of security with a lack of trust and feelings of anxiety. Experiencing anxiety can negatively impact women’s confidence in their birthing ability [93], which can continue during subsequent births [94, 95].

These findings illustrate the significance and importance of the presence of partners and professionals to women’s sense of security during birth [78–81]. A trusting relationship between health professionals and the woman giving birth seems to be essential for a mother’s sense of security and can strengthen her self-esteem [96]. It can be concluded that a woman’s feeling of security is not based exclusively on external competencies and caregivers but is essentially shaped by the nature of her relationship with health professionals. Therefore, midwives and obstetricians can significantly affect the woman’s sense of security via the nature of their relationship with the woman.

Regardless of the role of health professionals, the importance of the partner’s presence and support to the woman’s feeling of security is undeniable [78–81, 83, 84]. The importance of the father’s presence to the woman’s sense of security has already been mentioned in several studies concerning fathers’ childbirth experiences [49–51]. Having familiar person by their side appears to be important for helping women mentally deal with the uncertainty of the birth process. This role cannot be equated to the presence and support of health professionals given their various tasks and responsibilities. The woman’s need for her partner’s participation in the processes of pregnancy, childbirth and the postnatal period indicate that women understand the transitional phases as familial processes shared with their male partners.

For fathers, participation seems to be essential for their sense of security [84–86]. Further studies regarding fathers’ childbirth experiences have shown that having knowledge about childbirth and receiving information during the childbirth process increase the fathers’ sense of security and is accompanied by a sense of control [46, 97–99]. Fathers feel less involved and thus insecure if they do not receive needed information [98, 100]. On the other hand, fathers often try to hide their feelings of insecurity behind a confident and calm façade to support their female partner [101]. While women’s sense of control and confidence in themselves and their ability to give birth can create feelings of security, fathers’ sense of security reveals an exclusive dependence on their female partners and professionals. However, the presence and support of the caring professionals appears to be a factor connecting mothers and fathers. Support plays an essential role in developing confidence in caregivers and thus in creating a sense of security for both partners. Nevertheless, conclusions regarding the father’s security experiences are limited by the small number of included studies; only one study focused on the birth experience, and the remaining studies were restricted to the first postpartum week.

These findings indicate that a sense of security is characterised by an emotional, social and physical-medical dimension that has previously been described in some of the included studies [81, 83]. According to this viewpoint, the emotional dimension of feeling secure is represented by confidence and a sense of control and associated with freedom from anxiety [79–82, 86]. The social dimension of sense of security is reflected in the support and presence of the partner and in connections with other family members [78–81, 83, 84]. The physical-medical dimension is revealed on the one hand by the physical well-being of the woman and child [76, 79–84, 86] and on the other hand by the maternity care [76, 79, 82, 83, 86] and professional skills of the caregivers [77–80, 85, 86]. In turn, the perceived competence of the healthcare professionals plays an essential role in building a trusting relationship with the caregivers and can thus also be assigned to the emotional dimension of sense of security. The physical well-being of the mother and child seems to be another factor connecting the parents’ sense of security. Analogies to the perception and interpretation of the safety concept can be identified, and thus, from the women’s viewpoint, both concepts are not clearly separated from each other in the context of maternity care.

This integrative review synthesises a number of studies using diverse methodologies that were conducted in different health care systems. Methodological weaknesses regarding the recruitment or sample strategy were found in almost all of the qualitative studies. However, the included studies present a broad spectrum of internal and external factors influencing mothers’ and fathers’ sense of security and provide the opportunity for further specific recommendations. The results do not demonstrate an existing concept of sense of security or a consistent definition, and the findings of the influencing factors are mainly descriptive and less explanatory. Less is known about the causality between the security-creating determinants, which indicates that further qualitative studies exploring the constitution of sense of security in the context of childbirth are recommended. Furthermore, the present literature focuses primarily on the perspectives of pregnant women and mothers, whereas the fathers’ perspectives have been underresearched and are limited to the postpartum phase. Future studies are needed to exclusively investigate fathers’ perceptions of security experiences and needs during pregnancy or childbirth. The majority of participating women in several of the included studies were recruited from only the hospital setting. Further research considering different birth places and different modes of birth could better explain the influences of these factors on sense of security. Additionally, a common perspective of parents in the context of childbirth has not been investigated. Little is known about how parents experience and construct their own security in the context of childbirth. Further research is needed to fill this gap with respect to experiences of sense of security from the viewpoints of both parents.

These results summarise the perceptions of parents, in most cases of mothers from geographically and culturally diverse countries with different maternity care systems. Social and cultural backgrounds may influence mothers’ and fathers’ perceptions of security [31, 32]. Furthermore, there is a language-specific interpretative framework of the concept of security, as the term *security* is not distinguished from the concept of *safety* in German (*Sicherheit*) or Swedish (*Trygghet*). For this reason, obtaining feedback and knowledge of the experiences of parents within country-specific care systems is important and indicates the need for research in the local care context.

### Limitations

This review is potentially limited by the key phrases used in the database search. Therefore, not all of the relevant studies may have been found, as the search was limited to two biomedical and health science databases, one psychological science database and one German language social science database. In addition, the translation and interpretation of security and safety remain difficult and thus may lead to incomplete results. A further limitation is the integration of selected, security-relevant results from studies addressing different research questions. Finally, the selection of included articles was performed by only one researcher. To avoid possible bias, article selection should have been performed independently by two researchers. To minimise the methodological bias, the co-authors were continuously involved in the research process, and the thematic analysis results were discussed. Despite these limitations, this integrative literature review provides a broad overview of the knowledge related to parents’ sense of security during pregnancy, childbirth and the postnatal period and describes the factors that influence their feeling of security.

## Conclusions

This integrative review examined current knowledge of parents’ security experiences in the context of pregnancy, childbirth and the postnatal period. Previous findings from data analysis have indicated that the perception of security is highly complex and depends on multiple internal and external factors that can also differ between mothers and fathers. For mothers, the attribution of security as a basic need and central issue in pregnancy, childbirth and the postnatal period is a key result of the literature analysis. These results suggest a correlation between sense of security and feelings of control and confidence, showing the significance of partners’ and professionals’ support for a woman’s sense of security during birth. For fathers, participation seems to be essential for their sense of security. Research on the subjective experiences and perceptions of fathers pertaining to security is lacking. Little is known about the causality between security-related factors and sense of security. Further studies are needed to explore the constitution of sense of security in the context of childbirth. Additionally, further research focused on the experiences of security from the perspectives of fathers or both partners is necessary.

For both parents, the presence and support of midwives and other professional caregivers play important roles in developing confidence in the caregivers. A trusting relationship with midwives and other healthcare professionals is associated with parents’ sense of security. As such, midwives and other involved health professionals should be aware of their role in creating a sense of security among parents. Via targeted communication and advice, caregivers must determine what women and their partners need to feel secure during pregnancy, childbirth and the postnatal period. Based on these findings and a culture-specific understanding of security, midwives and other professional caregivers can effectively support parents with regard to their specific security needs and increase their chances for positive experiences during the transition to parenthood.
